# An Unusual Presentation of Generalized Tetanus Following Post-Exposure Vaccination Complicated by Acute Motor Sensory Axonal Neuropathy: A Case Report

**DOI:** 10.7759/cureus.72734

**Published:** 2024-10-30

**Authors:** Chanurdi K Wickramathunga, Philip Anpalahan, Thirunavukkarasu Thivakaran, Shehan Silva

**Affiliations:** 1 Medicine, Colombo South Teaching Hospital, Colombo, LKA; 2 Respiratory Medicine, National Hospital for Respiratory Diseases, Colombo, LKA; 3 Neurology, Colombo South Teaching Hospital, Colombo, LKA; 4 Medicine, Faculty of Medical Sciences, Colombo South Teaching Hospital - Kalubowila, University of Sri Jayewardenepura, Colombo, LKA

**Keywords:** acute motor sensory axonal neuropathy (amsan), neurotoxicity, rhabdomyolysis, tetanus, tetanus vaccination

## Abstract

Tetanus, caused by *Clostridium tetani*, remains a serious but preventable infection, with global incidence significantly reduced through widespread vaccination. We present the case of a 51-year-old man who developed severe tetanus following a minor nail prick injury, despite receiving tetanus toxoid and antibiotics. His condition rapidly progressed to trismus, hyperreflexia, continuous spasms, autonomic instability, and respiratory failure, requiring mechanical ventilation. A rare complication-acute motor sensory axonal neuropathy (AMSAN)-was identified, one of the first reported cases associated with tetanus. Despite aggressive treatment, the patient succumbed to multiorgan failure on day 22. This case highlights the high mortality of tetanus with short incubation periods, the challenge of managing autonomic instability, and the potential for rare complications like AMSAN, emphasizing the importance of timely recognition, intensive care, and management.

## Introduction

Tetanus is a life-threatening disease caused by *Clostridium tetani*, a Gram-positive, spore-forming bacillus commonly found in soil, capable of entering the body even through minor skin injuries. The prognosis of tetanus is closely tied to its incubation period (IP) of 3-21 days [1]; shorter intervals between injury and symptom onset are typically associated with more severe disease and higher mortality rates. Since 1990, the global incidence of tetanus has reduced by 88% in 30 years to less than 74,000 cases per year by 2019 due to efficient vaccination programs [2].

The pathophysiology of tetanus is primarily driven by the release of a potent exotoxin, tetanospasmin, which interferes with neurotransmitter release, leading to uncontrolled muscle contractions and severe autonomic dysfunction. While classic symptoms such as trismus and opisthotonus are well-documented, the disease can present with a wide spectrum of complications depending on the severity and speed of onset [1].

This article presents a rare case of severe tetanus complicated by acute motor sensory axonal neuropathy (AMSAN), a condition not previously associated with tetanus. We aim to explore the diagnostic and therapeutic challenges encountered in managing this case as well as the implications for future clinical practice in recognizing and treating tetanus and its rare complications.

## Case presentation

A previously healthy 51-year-old man presented with neck pain of insidious onset, which was gradually worsening over 24 hours and accompanied by difficulty in opening his mouth. He reported no fever, altered behavior, seizures, or other systemic symptoms. His medical and mental health histories were unremarkable. Five days before the presentation, he had sustained a minor nail prick injury, for which he received a tetanus toxoid injection within 24 hours. He also completed a five-day course of co-amoxiclav 625 mg thrice daily as prescribed.

**Figure 1:**
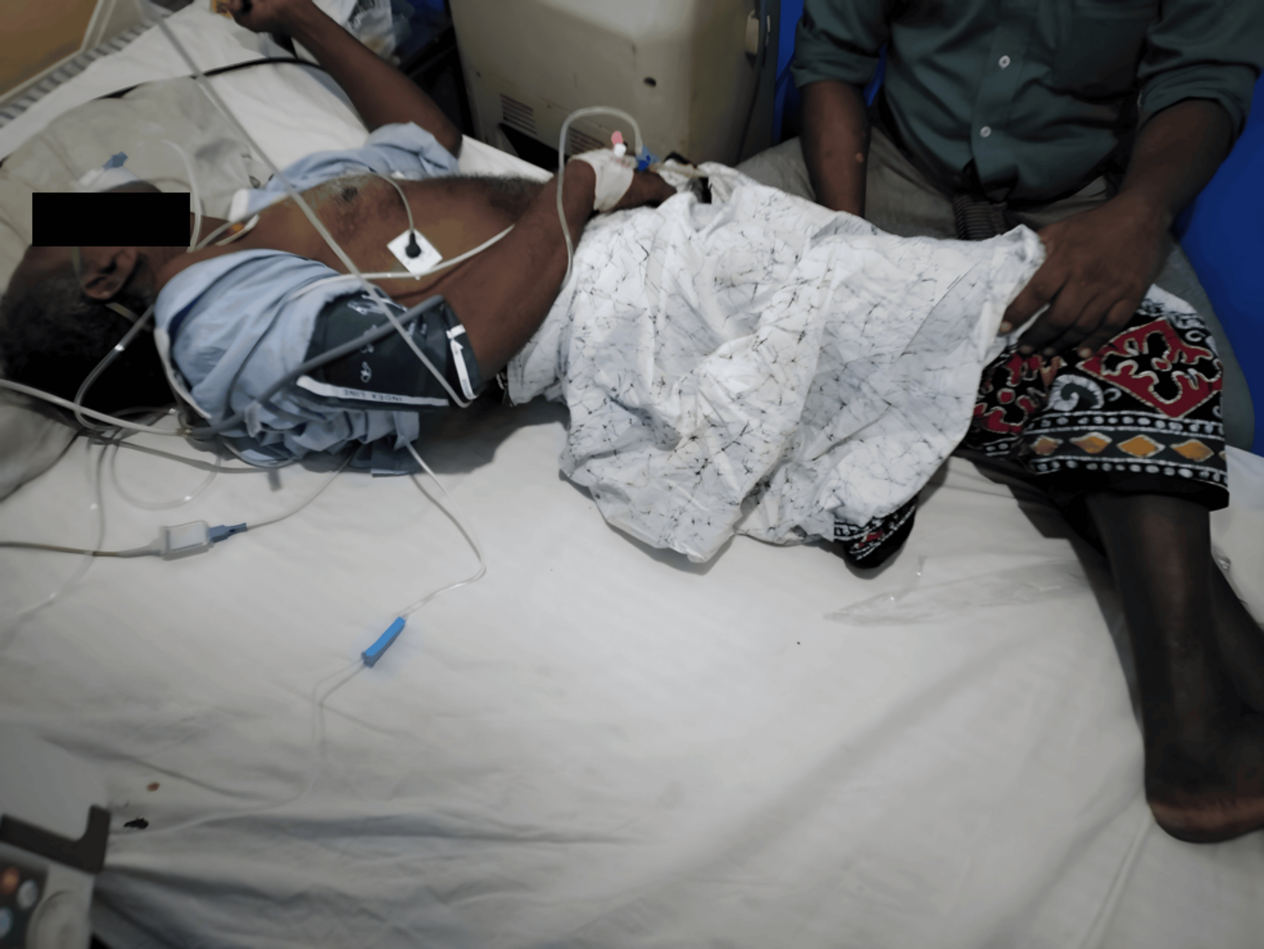
Opisthotonus positioning of the patient

The patient was alert and fully oriented (Glasgow Coma Scale (GCS) 15/15) despite exhibiting opisthotonus, trismus, and marked neck rigidity (Figure 1). His vital signs revealed a blood pressure of 150/80 mmHg and a pulse rate of 96 beats per minute. Respiratory examination was normal, and the injury site on the plantar surface of his left foot showed no signs of cellulitis or abscess. Neurological examination revealed generalized hyperreflexia with increased muscle tone.

The clinical findings were consistent with severe tetanus, and empirical treatment was initiated with intravenous benzylpenicillin (4 million units every six hours) and metronidazole (500 mg). He received 1500 IU of tetanus immunoglobulin injected around the wound site and an additional 3500 IU intramuscularly. Leucocyte count and differential count on admission were as follows: WBC, 18460/mcL; neutrophils, 14730/mcL; lymphocytes, 2450/mcL; monocytes, 870/mcL; eosinophils, 330/mcL; and basophils, 80/mcl.

The patient was managed in a low-stimulus, calm environment with supportive care including hydration, nutrition, and thromboprophylaxis. Despite these measures, he developed persistent tonic contractions and spasms, necessitating elective intubation. Initial spasm control was attempted with intravenous midazolam, followed by intravenous magnesium sulfate (MgSO_4_) titrated to 2.5 mg/h. Due to inadequate response, additional agents including baclofen, intravenous propofol, phenytoin, and tizanidine were administered. Sedation was maintained with vecuronium, and the patient underwent surgical debridement following intubation.

**Figure 2:**
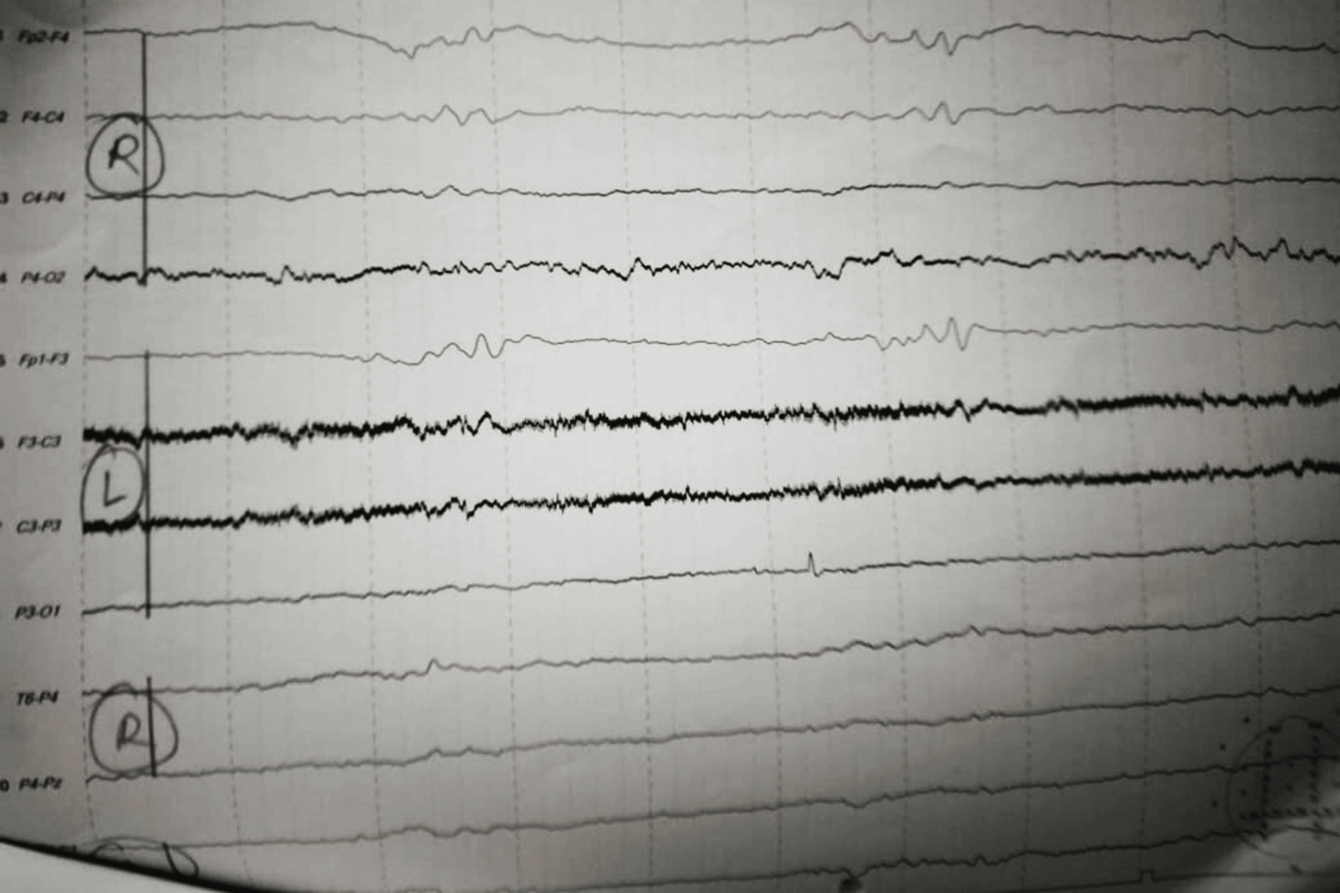
EEG on day 19 EEG: Electroencephalogram.

Retroviral studies were negative, and a non-contrast CT scan of the brain was unremarkable, with no signs of encephalopathy or encephalitis observed on the initial electroencephalogram (EEG). A repeat EEG done on day 19 demonstrated a severe form of encephalopathy characterized by burst suppression (Figure 2).

Cerebrospinal fluid analysis, performed twice on the fourth and 18th days of the illness, was normal, and anti-glutamic acid decarboxylase (GAD) antibodies were negative. A brain MRI with contrast was normal. The nerve conduction study revealed AMSAN for which a five-day intravenous immunoglobulin was administered (Table 1).

**Table 1:** Nerve conduction study

Nerve and Site	Latency	Amplitude	Segment	Latency Difference (ms)	Distance (mm)	Conduction Velocity (m/s)
Sensory Nerve Conduction						
Sural R						
Lower leg	-	Not recordable	Ankle – lower leg	-	-	-
Sural L						
Lower leg	-	Not recordable	Ankle – lower leg	-	-	-
Median L Ulnar Transcarpal Comparison						
Median palm	-	Not recordable	Median palm – wrist	-	-	-
Ulnar palm	-	Not recordable	Ulnar palm – wrist	-	-	-
			Median palm – ulnar palm	-	-	-
Motor Nerve Conduction						
Peroneal R						
Ankle	-	Not recordable	Extensor digitorum brevis – ankle	-	-	-
Fibula (head)	-		Ankle – fibula (head)	-	-	-
Tibial R						
Ankle	-	Not recordable	Abductor hallucis – ankle	-	-	-
Peroneal L						
Ankle	-	Not recordable	Extensor digitorum brevis – ankle	-	-	-
Fibula (head)	-		Ankle – fibula (head)	-	-	-
Tibial L						
Ankle	-	Not recordable	Abductor hallucis – ankle	-	-	-
Ulna R						
Wrist	3.5 ms	1.5 mV	Abductor digiti minimi – wrist	3.5 ms	-	-
Below elbow	8.9 ms	1.1 mV	Wrist – below the elbow	5.4 mw	260 mm	48 m/s
Median L						
Wrist	-	Not recordable	Abductor pollicis brevis – wrist	-	-	-
Elbow	-		Wrist – elbow	-	-	-
Ulna L						
Wrist	2.0 ms	0.4 mV	Abductor digiti minimi – wrist	2.0 ms	-	-
Below L	8.2 ms	0.3 mV	Wrist – below the elbow	6.2 ms	260mm	45 m/s

The patient subsequently developed significant fluctuations in blood pressure and heart rate, indicative of autonomic instability. This was managed with intravenous MgSO_4_ infusion, which was titrated until the patellar reflex was absent. Despite increasing the dosage, the spasms persisted.

The patient developed rhabdomyolysis, which was managed with forced alkaline diuresis and hemodialysis. He subsequently experienced complications, including ventilator-associated pneumonia and septicemia with multiorgan failure, which were treated with intravenous antibiotics tailored to sensitivity patterns, along with appropriate organ support. Despite these interventions, he passed away on day 22. A post-mortem examination revealed no structural abnormalities, such as tumors or brain abscesses.

## Discussion

Our patient exhibited severe trismus, generalized spasticity, prolonged spasms, tachycardia, and autonomic dysfunction, meeting the criteria for severe generalized tetanus as outlined by the Ablett criteria [3]. Even minor injuries can lead to tetanus, with up to 30% of patients having a recognizable portal of entry [4]. In low-prevalence regions, outcomes are often poor due to delays in diagnosis [5]. Failure to promptly recognize and treat the condition can be fatal. Timely administration of tetanus toxoid and immunoglobulin is crucial in reducing disease severity. The unusual lack of response to post-exposure prophylaxis in this case may be due to factors such as a more virulent bacterial strain, diminished vaccine potency, or immunosuppression from antibiotics or other causes [6].

The duration of the IP and the time to symptom onset are crucial for prognosis, with shorter incubation periods often resulting in more severe manifestations [7]. The IP reflects the time it takes for the toxin to reach the central nervous system and the quantity that has entered the body. In our patient, with an IP of five days, generalized spasms developed just a few hours after the onset of trismus. He had a Dakar score of 5 (IP: five days, onset of symptoms < 24 hours, along with spasms, fever, and tachycardia), indicating severe disease with a mortality rate exceeding 50% [7].

In our case, benzylpenicillin was initially administered but later escalated to intravenous ceftriaxone due to the neuro-hyperexcitability associated with benzylpenicillin [8]. This agent is known to have the highest epileptogenic potential, independent of cerebrospinal fluid concentrations [9]. The mechanism is thought to involve the inhibition of gamma-aminobutyric acid (GABA) transmission, as the beta-lactam penicillin ring structure closely mimics that of GABA neurotransmitters [10].

A unique finding in this patient was the development of AMSAN, which is not a typical feature of tetanus. The neuropathy was likely axonal in nature, as the short duration of the illness makes secondary axonal neuropathy following demyelination less probable. This phenomenon could be related to the tetanus vaccination, or tetanus itself may have caused this form of neuropathy. Evidence in the literature suggests that patients can develop neuropathy, with weakness and sensory loss resembling peripheral neuropathy or Guillain-Barré syndrome, either following tetanus toxoid administration or as a complication of tetanus infection [11,12]. However, this is a rare occurrence of severe AMSAN associated with tetanus. AMSAN has previously been reported with infections such as COVID-19, mycoplasma, HIV, cytomegalovirus, and Epstein-Barr virus [13-16].

Autonomic instability poses a significant challenge in managing the patient and is recognized as a feature of poor prognosis. Although high-dose intravenous MgSO_4_ has been shown to alleviate spasms and autonomic instability and reduce the need for mechanical ventilation in mild and moderate cases of tetanus [17], its efficacy in severe tetanus is less clear. In severe cases, MgSO_4_ can manage both autonomic dysfunction and muscle spasms but does not reduce the need for mechanical ventilation [18]. To avoid MgSO_4_ overdose, we closely monitored the patellar reflex and maintained serum magnesium levels below 4 mmol/L.

## Conclusions

This case illustrates the severe nature of tetanus, where even minor injuries can lead to life-threatening outcomes despite post-exposure prophylaxis. Delayed or ineffective response to prophylaxis may contribute to disease severity. Our patient developed severe generalized tetanus, complicated by AMSAN, a rare but severe neurological complication not previously associated with tetanus. This is one of the rare cases reporting AMSAN associated with tetanus, raising important considerations for future clinical practice. This highlights the need for clinicians to recognize potential complications like AMSAN and the importance of early, aggressive treatment. Despite interventions, the prognosis remains poor in severe cases. Further research is essential to deepen our understanding of the mechanisms underlying non-responsiveness to prophylaxis and the neurological complications of tetanus, which will aid in improving management strategies for such cases in the future.

## References

[1] Hassel B (2013). Tetanus: pathophysiology, treatment, and the possibility of using botulinum toxin against tetanus-induced rigidity and spasms. Toxins (Basel).

[2] Li J, Liu Z, Yu C, Tan K, Gui S, Zhang S, Shen Y (2023). Global epidemiology and burden of tetanus from 1990 to 2019: a systematic analysis for the Global Burden of Disease Study 2019. Int J Infect Dis.

[3] Lu P, Ghiasi S, Hagenah J (2022). Classification of tetanus severity in intensive-care settings for low-income countries using wearable sensing. Sensors (Basel).

[4] (2024). Morbidity and mortality weekly report (MMWR). Morbidity and Mortality Weekly Report.

[5] Rodrigo C, Fernando D, Rajapakse S (2014). Pharmacological management of tetanus: an evidence-based review. Crit Care.

[6] Krüger S, Seyfarth M, Sack K, Kreft B (1999). Defective immune response to tetanus toxoid in hemodialysis patients and its association with diphtheria vaccination. Vaccine.

[7] Thwaites CL, Yen LM, Glover C (2006). Predicting the clinical outcome of tetanus: the tetanus severity score. Trop Med Int Health.

[8] Johnson H, Walker AE (1945). Intraventricular penicillin: a note of warning. J Am Med Assoc.

[9] Kolb R, Gogolák G, Huck S, Jaschek I, Stumpf C (1976). Neurotoxicity and CSF level of three penicillins. Arch Int Pharmacodyn Ther.

[10] Schliamser SE, Cars O, Norrby SR (1991). Neurotoxicity of beta-lactam antibiotics: predisposing factors and pathogenesis. J Antimicrob Chemother.

[11] Pollard JD, Selby G (1978). Relapsing neuropathy due to tetanus toxoid. Report of a case. J Neurol Sci.

[12] Im SJ, Hwang YS, Park HY, Cheong JS, Lee HS, Lee JH (2017). Guillain-Barre syndrome after generalized tetanus infection. Annals of Clinical Neurophysiology.

[13] Geng N, Wang P, Zhang Y (2023). Acute motor-sensory axonal polyneuropathy variant of Guillain-Barré syndrome with a thalamic lesion and COVID-19: a case report and discussion on mechanism. Front Neurol.

[14] Gupta R, Gupta A, Goyal V, Guleria R, Kumar A (2005). Mycoplasma pneumonia associated with rhabdomyolysis and the Guillain-Barre syndrome. Indian J Chest Dis Allied Sci.

[15] Vidal JE, Guedes BF, Gomes HR, Mendonça RH (2022). Guillain-Barré syndrome spectrum as manifestation of HIV-related immune reconstitution inflammatory syndrome: case report and literature review. Braz J Infect Dis.

[16] Taheraghdam A, Pourkhanjar P, Talebi M, Bonyadi M, Pashapour A, Sharifipour E, Rikhtegar R (2014). Correlations between cytomegalovirus, Epstein-Barr virus, anti-ganglioside antibodies, electrodiagnostic findings and functional status in Guillain-Barré syndrome. Iran J Neurol.

[17] Thwaites C, Yen L, Loan H (2006). Magnesium sulphate for treatment of severe tetanus: a randomised controlled trial. Lancet.

[18] Karanikolas M, Velissaris D, Marangos M, Karamouzos V, Fligou F, Filos KS (2010). Prolonged high-dose intravenous magnesium therapy for severe tetanus in the intensive care unit: a case series. J Med Case Rep.

